# Roles of the Translation Initiation Factor eIF2α Phosphorylation in Cell Structure and Function

**DOI:** 10.1247/csf.20013

**Published:** 2020-04-29

**Authors:** Sung Hoon Back

**Affiliations:** 1 School of Biological Sciences, University of Ulsan, Ulsan 44610, Korea

**Keywords:** eIF2α phosphorylation, Translation, Unfolded Protein Response, Reactive Oxygen Species, Mitochondria

## Abstract

It is often assumed that α-subunit phosphorylation of the eukaryotic translation initiation factor 2 (eIF2) complex is just a mechanism to control protein synthesis. However, eIF2α phosphorylation induced by multiple kinases can recognize various intracellular and extracellular stress conditions, and it is involved in various other cellular processes beyond protein synthesis. This review introduces the roles of eIF2α phosphorylation in translational regulation, the generation of reactive oxygen species, changes in mitochondria structure and shape, and mitochondrial retrograde signaling pathways in response to diverse stress conditions.

## Roles of eIF2α phosphorylation in translational regulation during diverse stress conditions

### Suppression of global translation and eIF2α phosphorylation

**Fig. 1:**
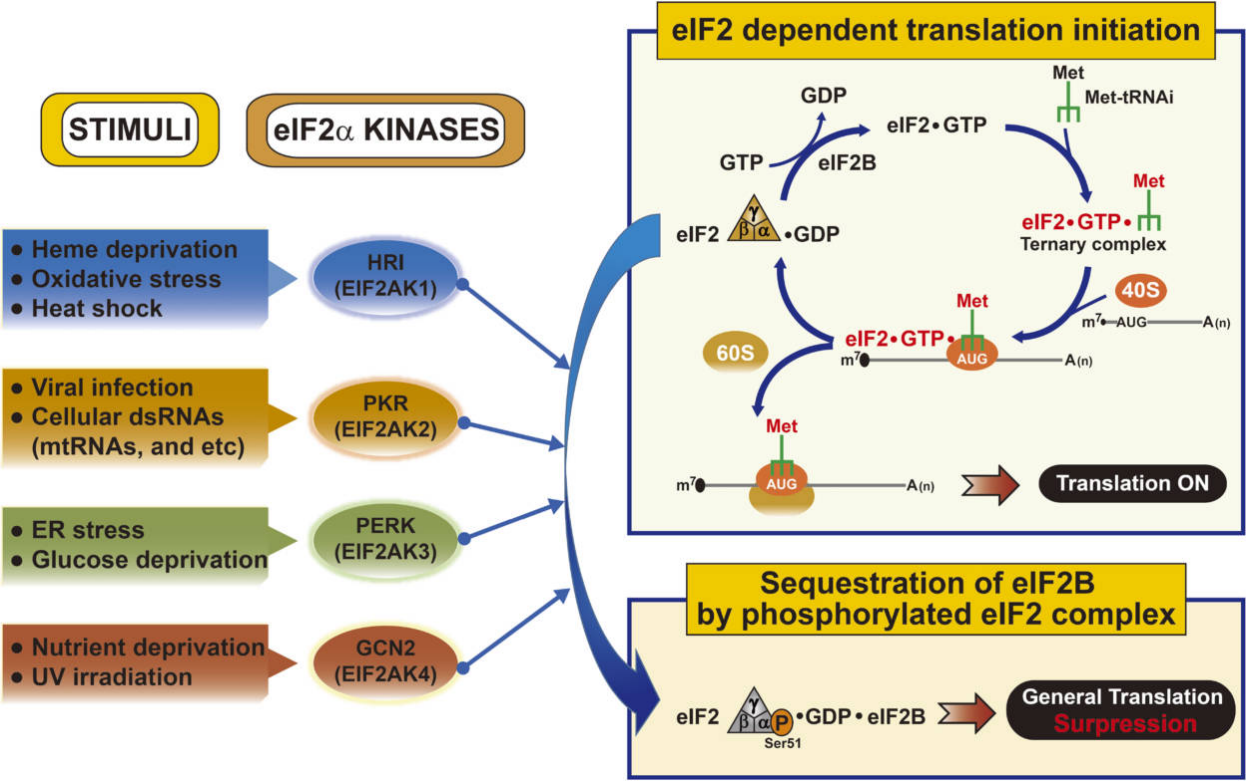
The molecular mechanism of eIF2α phosphorylation-dependent translation initiation controlled by multiple eIF2α kinases during various stress conditions.

In translation initiation, delivery of methionyl-initiator tRNA (Met-tRNA_i_^Met^) to the 40S ribosomal subunit is mediated by the GTP-bound eukaryotic translation initiation factor 2 (eIF2) complex, which consists of α, β, and γ subunits (Proud, 2005). The binding of GTP to eIF2 is the rate-limiting step for Met-tRNA_i_^Met^ delivery and ultimately for protein synthesis (Sonenberg and Hinnebusch, 2009; Wek *et al.*, 2006). Binding of GTP to eIF2 is mediated by guanine exchange activity (GEF) of the eIF2B complex (Wortham and Proud, 2015). In cells exposed to diverse stress conditions such as heme deprivation, viral infection, endoplasmic reticulum (ER) stress, and amino acid starvation, four mammalian protein kinases—HRI/EIF2AK1 (eIF2α kinase 1), PKR/ EIF2AK2 (eIF2α kinase 2), PERK/EIF2AK3 (eIF2α kinase 3), and GCN2/EIF2AK4 (eIF2α kinase 4)—phosphorylate serine 51 of the α subunit of the eIF2 complex (Donnelly *et al.*, 2013; Wek *et al.*, 2006) (Fig. 1). Recently, Taniuchi *et al.* tested for additional unknown eIF2α kinases in vertebrates using quadruple knockout cells for all four known eIF2α kinases in 12 different intracellular and extracellular stress conditions, with the exception of heme deprivation, viral infection, and amino acid starvation (Taniuchi *et al.*, 2016). The authors reported finding no additional eIF2α kinases, although the possibility remains that an unknown cell- or stress-specific eIF2α kinase is present in vertebrates. The phosphorylation of eIF2α inhibits the exchange of GDP for GTP by eIF2B on the eIF2 complex and thereby prevents formation of the eIF2-GTP-Met-tRNAi ternary complex, and therefore delivery of Met-tRNA_i_^Met^ to the 40S ribosomal subunit (Wortham and Proud, 2015). Regardless of the type of stimulus, eIF2α phosphorylation causes an identical reduction in global protein synthesis (Donnelly *et al.*, 2013; Sonenberg and Hinnebusch, 2009; Wek *et al.*, 2006) (Fig. 1). It may therefore permit cells to conserve deficient resources and/or prevent further accumulation of stress-causing materials.

### Preferential translation of various specific mRNAs and eIF2α phosphorylation

**Fig. 2:**
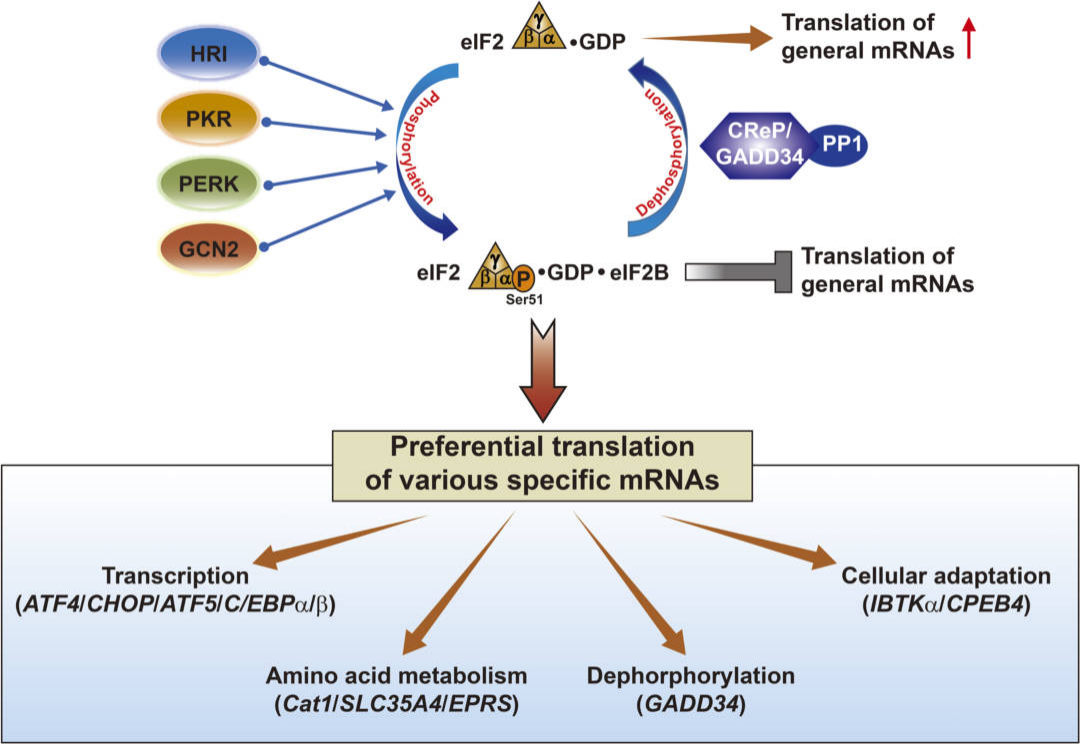
Various specific mRNAs preferentially translated in the conditions of increased eIF2α phosphorylation. Dephosphorylation of eIF2α is mediated by the catalytic subunit of protein phosphatase 1 (PP1) in complex with either of two regulatory subunits GADD34 and CReP (constitutive repressor of eIF2α phosphorylation), respectively (Harding *et al.*, 2009).

Global translation reduction is not a unique cellular response to stresses induced by eIF2α phosphorylation. Paradoxically, increased eIF2α phosphorylation promotes translation of diverse selected mRNAs that are inefficiently translated in the absence of stress. Preferentially translated gene transcripts include several basic leucine zipper transcription factors [ATF4 (CREB2) (Kilberg *et al.*, 2009), CHOP (DDIT3/GADD153) (Palam *et al.*, 2011), ATF5 (Zhou *et al.*, 2008), and C/EBPα and β (Calkhoven *et al.*, 2000; Li *et al.*, 2008)], amino acid metabolism-related genes [cationic amino acid transporter (Cat-1)] (Fernandez *et al.*, 2002), probable UDP-sugar transporter protein SLC35A4 (SLC35A4) (Andreev *et al.*, 2015), bifunctional glutamate/proline-tRNA ligase (EPRS) (Young *et al.*, 2016), a protein phosphatase 1 (PP1) regulatory subunit responsible for eIF2α dephosphorylation [GADD34 (PPP1R15A)] (Lee *et al.*, 2009), and other protective genes [IBTKα (Baird *et al.*, 2014) and CPEB4 (Maillo *et al.*, 2017)] (Fig. 2). Most proteins produced from preferentially translated mRNAs play roles in adapting to cellular stress and restoring homeostasis (Young and Wek, 2016). However, if cellular stress cannot be overcome, transcription factors such as ATF4 and CHOP can induce cell death through increased protein synthesis, resulting in oxidative stress and ATP depletion (Han *et al.*, 2013) or regulated expression of downstream pro/anti-apoptotic target genes (such as DR5, TRB3, Bim, and Bcl-2) (Tabas and Ron, 2011). Furthermore, sustained translational repression by eIF2α phosphorylation is also reportedly deleterious to cells exposed to chronic stress in cognitive and neurodegenerative disorders because repression inhibits normal protein production (Moreno *et al.*, 2012; Zhu *et al.*, 2019).

All the transcripts described above have short upstream open reading frames (uORFs) located in the 5'-untranslated region (5'-UTR) of the mRNA. They are preferentially translated through uORF-mediated mechanisms during diverse stressful conditions in which eIF2α phosphorylation is induced by four eIF2α kinases (Pakos-Zebrucka *et al.*, 2016; Young and Wek, 2016). The uORFs of the transcripts described above can affect downstream coding sequence (CDS) translation in multiple ways. Translation can be affected through the promotion of ribosome re-initiation at the downstream CDS after the uORF translation, the stalling of ribosome elongation while translating the uORF, ribosome dissociation after translation of the uORF, ribosome translation past the CDS start codon resulting in no translation initiation at the CDS, or the bypassing of the uORF by the ribosome (Hinnebusch *et al.*, 2016; Young and Wek, 2016). If a transcript has uORFs that promote ribosome re-initiation or bypass in response to environmental stresses, enhancement of translation initiation will occur at the downstream CDS but low or no translation of the CDS will occur under normal conditions. Thus, eIF2α phosphorylation–dependent translations of the mRNAs described above can be achieved through the proper mixing and matching of uORFs in their 5'-UTRs. However, specific uORF configurations and working mechanisms in each gene were obtained through evolutionary adaptation to relevant stresses. Individual genes may have unique features of uORFs that permit preferential translation in response to eIF2α phosphorylation, implying that there are multiple mechanisms asserted by diverse uORFs. Detailed working mechanisms of uORFs are well described in two reference papers (Hinnebusch *et al.*, 2016; Young and Wek, 2016).

## Roles of eIF2α phosphorylation beyond translation regulation

Although the biochemical mechanism of translation inhibition mediated by eIF2α phosphorylation is well understood, its downstream physiological consequences are less clear at the cellular level. Upon ER stress, viral infection, and other cellular stresses, four eIF2α kinases phosphorylate eIF2α, which leads to transient attenuation of global protein synthesis and transcriptional induction through preferential translation of selected transcription factor genes (such as ATF4, ATF5, CHOP, and C/EBPα and β) (Donnelly *et al.*, 2013; Wek *et al.*, 2006). As the common point of convergence for all the stress stimuli, eIF2α phosphorylation activates an evolutionarily conserved signaling pathway, known as integrated stress response (ISR) to restore cellular homeostasis (Pakos-Zebrucka *et al.*, 2016; Ron, 2002; Wek *et al.*, 2006). Many studies have reported that eIF2α phosphorylation regulates various cellular processes. This review summarizes the roles of eIF2α phosphorylation in oxidative stress and mitochondria function.

### Reactive oxygen species and eIF2α phosphorylation

**Fig. 3:**
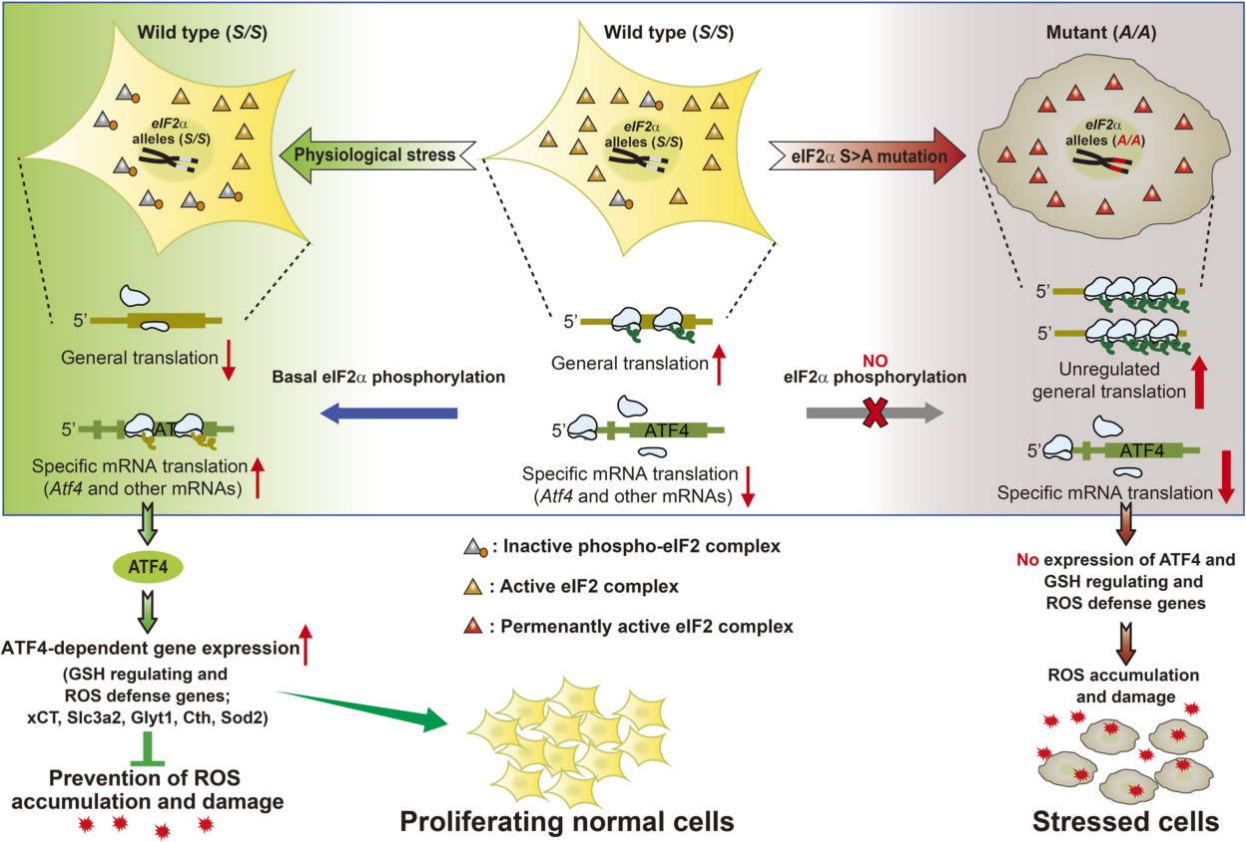
Oxidative stress defense mechanism mediated by basal eIF2α phosphorylation and ATF4 expression and ROS accumulation mechanism induced by eIF2α phosphorylation deficiency.

Although eIF2α phosphorylation can promptly and robustly change in response to stress, a basal level of eIF2α phosphorylation has also been observed in cells cultured *in vitro* (Rajesh *et al.*, 2013; Scheuner *et al.*, 2001) and *in vivo* (Hussain and Ramaiah, 2007; Lewerenz and Maher, 2009). Multiple studies indicate that basal eIF2α phosphorylation prevents oxidative stress by modulating antioxidant levels under normal conditions which may have physiological stress (Choi *et al.*, 2017; Harding *et al.*, 2003; Lewerenz and Maher, 2009; Rajesh *et al.*, 2013). Genetic inactivation of eIF2α phosphorylation in mouse embryonic fibroblasts (MEFs) and immortalized hepatocytes lead to increased intracellular ROS levels (Choi *et al.*, 2017; Rajesh *et al.*, 2013). Furthermore, eIF2α phosphorylation deficiency impairs proliferation and induces premature senescence, which can be prevented by anti-oxidant treatment (Rajesh *et al.*, 2013). Consistent with ROS accumulation in eIF2α phosphorylation-deficient (*A/A*, homozygous Ser51Ala mutant eIF2α alleles) MEFs, lower average levels of glutathione (GSH), a tripeptide antioxidant that contains L-cysteine, L-glutamic acid, and glycine, can be expected, compared with wild-type (*S/S*, homozygous Ser51 eIF2α alleles) MEFs. Reduced GSH levels in *A/A* cells are related to impaired glutathione metabolism because of decreased eIF2α phosphorylation–dependent ATF4 translation and subsequent downregulation of the light chain, xCT (encoded by the Slc7a11 gene), of the X_c_^-^ cystine/glutamate exchanger (Harding *et al.*, 2003; Lewerenz and Maher, 2009). Several xCT promoter studies have reported that xCT transcription is induced by ATF4 binding to amino acid response elements in its promoter during ER stress or amino acid deprivation (Lewerenz and Maher, 2009; Sato *et al.*, 2004). Moreover, ATF4-deficient cells are impaired in the ability to express genes involved in GSH biosynthesis and anti-oxidative stress. For GSH biosynthesis, these include the heavy chain (Slc3a2) of the X_c_^-^ cystine/glutamate exchanger, the glycine transporter (Glyt1), and cystathionine γ-lyase (Cth) (Fusakio *et al.*, 2016; Harding *et al.*, 2003). In response to anti-oxidative stress, the absence of ATF4 reduces expression of mitochondrial superoxide dismutase (Sod2) (Fusakio *et al.*, 2016). Loss of ATF4 therefore results in enhanced oxidative damage (Fusakio *et al.*, 2016; Harding *et al.*, 2003). In line with these results, both eIF2α phosphorylation and ATF4-deficient cells were found to be highly sensitive to oxidative stresses (Choi *et al.*, 2017; Harding *et al.*, 2003). These studies suggest that small but sufficient expression of antioxidant genes by basal eIF2α phosphorylation and its downstream target ATF4 expression are necessary to prevent ROS damage, even in normal conditions (Fig. 3).

As in the case of the described *in vitro* studies, basal eIF2α phosphorylation is required to protect specialized secretory cells, including pancreatic β cells, from oxidative stress *in vivo* (Back *et al.*, 2009). The acute removal of β cell–specific eIF2α phosphorylation raised uncontrolled translation, ROS accumulation, dysfunction, and death in animal model β cells. The phosphorylation of eIF2α coordinately attenuates translation of glucose-regulated proinsulin mRNA, prevents oxidative stress, and optimizes ER protein-folding in support of insulin production in animal model β cells (Back *et al.*, 2009). However, it is not clear whether reduced expression of ATF4 and its downstream anti-oxidant genes are responsible for observed phenotypes in eIF2α phosphorylation-deficient β cells because the study of whole-body *ATF4* knockout mice has shown that ATF4 is not required to preserve β cell function (Back *et al.*, 2009), and further studies using β cell–specific *ATF4* knockout mouse models are required to clarify the contribution of ATF4 against oxidative stress in β cells.

**Fig. 4:**
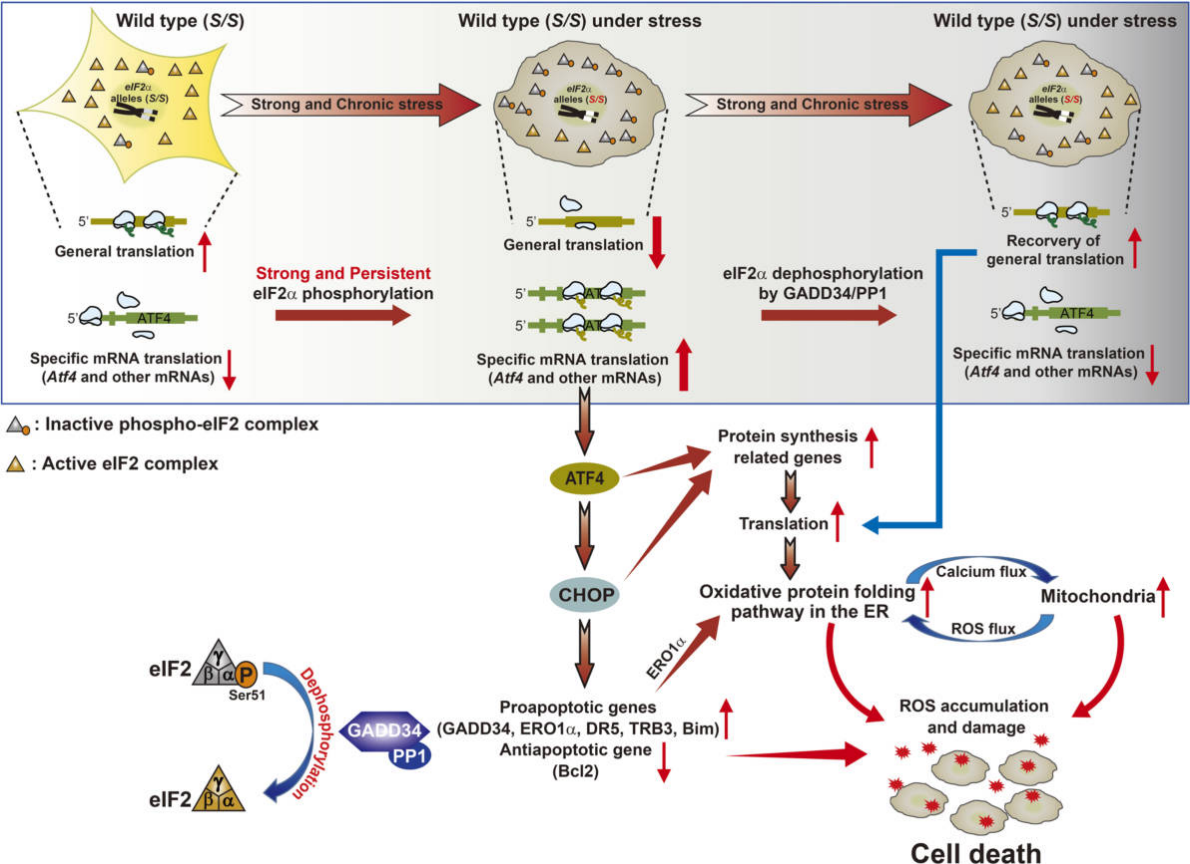
Oxidative stress and cell death pathways induced by chronic ISR pathway activation.

However, the eIF2α phosphorylation-ISR pathway induced by PERK works as an acute or chronic ER stress–induced cell-death pathway (Tabas and Ron, 2011; Wang and Kaufman, 2014). During ER stress, PERK-mediated eIF2α phosphorylation increases the expression of ATF4 and its key downstream target, CHOP (Ron and Walter, 2007). In addition, induced ATF4 and CHOP transcription factors increase transcription of the growth-arrest and DNA damage–inducible protein 34 (GADD34/PPP1R15A) to direct eIF2α dephosphorylation and restore global mRNA translation (Back and Kaufman, 2012; Ron and Walter, 2007). Recently, Han *et al.* suggested that persistent and strong expression of ATF4 and CHOP increase protein synthesis in GADD34-dependent and -independent manners and cause oxidative stress and cell death (Han *et al.*, 2013) (Fig. 4). For GADD34-independent protein synthesis, ATF4 and CHOP act together to upregulate target genes encoding functions in protein synthesis (such as multiple aminoacyl-tRNA synthetases, ribosomal subunits, and eukaryotic translation initiation factor subunits) to restore general mRNA translation under ER stress (Han *et al.*, 2013). Increased protein synthesis will then push a cell to generate damaging ROS through related cellular organelles, such as the ER and mitochondria (Han *et al.*, 2013). In the ER, ROS are produced as by-products while electrons shuttle through protein disulfide isomerase and ER oxidoreductin 1α (ERO1α) to O_2_ in oxidative protein-folding pathways for disulfide bond formation (Back and Kaufman, 2012). ERO1α may also hyperoxidize the ER lumen and activate the inositol trisphosphate receptor, causing a release of Ca^2+^ from the ER (Li *et al.*, 2009). Next, the increase in mitochondrial Ca^2+^ uptake induces a mitochondrial Ca^2+^ overload, which leads to enhanced ROS generation by stimulating the TCA cycle and oxidative phosphorylation (Brookes *et al.*, 2004). In addition, CHOP is a well-known proapoptotic transcription factor that can induce several proapoptotic genes (ERO1α, GADD34, DR5, TRB3, and Bim) or repress an antiapoptotic gene (Bcl2) (Fig. 4). Detailed molecular mechanisms of CHOP-induced apoptosis have been fully described (Iurlaro and Munoz-Pinedo, 2016; Tabas and Ron, 2011).

Collectively, both hypo- and hyper-eIF2α phosphorylation are responsible for cellular ROS accumulation and cause cell death because of hypo- or hyper-expression of downstream target genes. The level of eIF2α phosphorylation should therefore be in a balanced range to promote successful adaptation to cellular stress (Wek and Anthony, 2009).

### Mitochondrial structure and shape and eIF2α phosphorylation

**Fig. 5:**
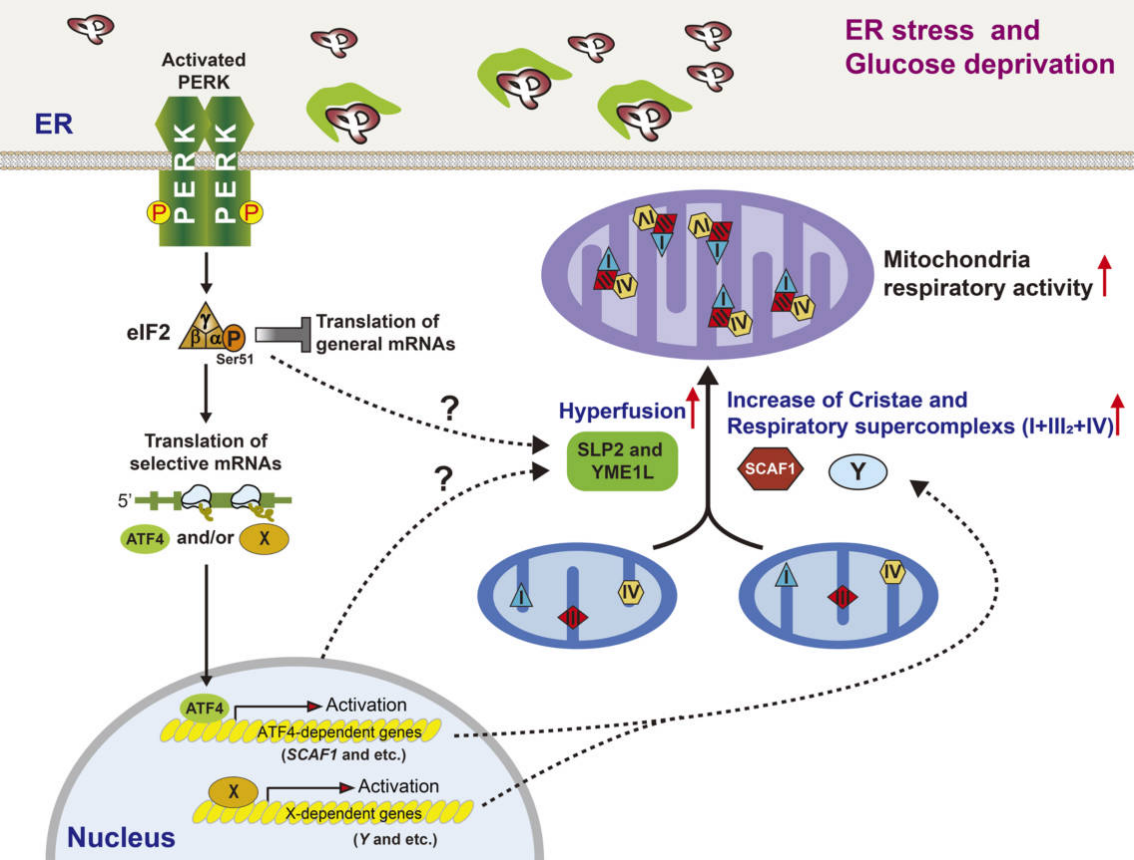
PERK-eIF2α phosphorylation-dependent mitochondria hyperfusion and respiratory supercomplex formation during ER stress and glucose deprivation conditions.

Although eIF2α phosphorylation appears to affect only cytosolic translation initiation, the effect is not restricted to translation because eIF2α kinases and eIF2α-dependent genes are involved in various cellular responses from multiple places in a cell. Recent reports suggest that mitochondria can be regulated through eIF2α phosphorylation in response to diverse stresses (Back *et al.*, 2009; Balsa *et al.*, 2019; Lebeau *et al.*, 2018; Zheng *et al.*, 2012). The structural role of PERK in stabilizing ER-mitochondrial contacts (Munoz *et al.*, 2013) implies that PERK-deficient cells suffer from defects in mitochondrial functions. PERK silencing reportedly stimulated mitochondrial respiration, whereas overexpressing PERK suppressed mitochondrial respiration and led to fragmented or rounded mitochondria (Munoz *et al.*, 2013). However, the eIF2α phosphorylation requirement was not checked in Munoz’s results. In addition, opposite effects of PERK signaling have been reported in mitochondrial respiration and morphology (Back *et al.*, 2009; Balsa *et al.*, 2019; Lebeau *et al.*, 2018; Zheng *et al.*, 2012). Although further studies are required to clarify the discrepancies in the described reports, PERK signaling–deficient cells show perturbed responses to ER and nutrient stress, including defects in respiratory supercomplex formation, increased mitochondrial cristae (Balsa *et al.*, 2019) and mitochondrial hyperfusion (Lebeau *et al.*, 2018), all of which are important to mitochondrial integrity and homeostasis. Recently, the functional organization of electron transport chain (ETC) complexes was explained by a plasticity model, in which individual ETC complexes coexist with superassembled structures (including I+III_2_+IV_1_, I+III_2_, and III_2_+IV_1–2_) called supercomplexes (SCs) (Cogliati *et al.*, 2016). This organization of complexes allows for more efficient transportation of electrons. Balsa *et al.* suggested that mitochondrial respiratory activity is elevated through increased cristae formation and SC levels to satisfy energetic and metabolic demands during glucose deprivation and ER stress (Balsa *et al.*, 2019). These structural changes are driven by activation of the PERK signaling pathway. The PERK-eIF2α axis transcriptionally controls expression of supercomplex assembly factor 1 (SCAF1/COX7A2L) through one of its downstream targets, which is translationally expressed ATF4 transcription factor (Balsa *et al.*, 2019) (Fig. 5). Because SCAF1 mediates the interaction between CIII and CIV, its presence determines the formation of two SCs I+III+IV (the respirasome) and III+IV (Lapuente-Brun *et al.*, 2013). As with SCAF1 null cells, PERK, eIF2α phosphorylation, or ATF4-deficient cells displayed bioenergetics defects such as reduced SCs, mitochondrial respiration, and ATP levels and then compromised their proliferation during ER stress and glucose-deprived conditions (Balsa *et al.*, 2019). SC formation and proliferation were restored in ATF4 null cells, but not in PERK null cells, by overexpressing SCAF1, suggesting that SCAF1 may not be the only factor that promotes assembly of SCs through the PERK-eIF2α axis during ER and nutrient stress (Fig. 5). Further investigation is required to identify other assembly factors. In addition, whether other eIF2α kinases-eIF2α pathways can remodel mitochondrial ultrastructures to activate oxidative phosphorylation in the response of different cellular stresses remain an open question.

Mitochondrial elongation reportedly protects mitochondria from autophagic degradation during nutrient starvation (Gomes *et al.*, 2011; Rambold *et al.*, 2011). In parallel with increased SC assembly, the PERK-eIF2α axis also promotes stress-induced mitochondrial hyperfusion to protect mitochondria during ER stress (Lebeau *et al.*, 2018). Hyperfusion protects mitochondria from pathologic fragmentation and increases metabolism to facilitate recovery from acute ER stress. Unlike SC assembly, PERK-mediated mitochondrial elongation does not require ATF4 transcriptional activity but does need eIF2α phosphorylation–dependent translational attenuation (Fig. 5). Furthermore, ER stress–dependent elongation requires SLP2 and YME1L in a mitochondrial large-protease complex that includes the membrane scaffold SLP2, rhomboid protease PARL, and ATP-dependent mitochondrial inner membrane protease YME1L (Lebeau *et al.*, 2018), reflecting the importance of degrading a short-lived mitochondrial protein in elongation. No known molecular mechanism explains why PERK-eIF2α phosphorylation needs SLP2 and YME1L proteins, although they are important factors in elongation during ER stress (Fig. 5). The involvement of GCN2-eIF2α pathways in mitochondrial elongation induced by amino acid starvation (Gomes *et al.*, 2011; Rambold *et al.*, 2011) should also be studied.

### Mitochondrial retrograde signaling pathways and eIF2α phosphorylation

Mitochondria communicate with the cell through mitochondrial retrograde signaling pathways (MRSP), which signal mitochondrial dysfunction to the cytosol or nucleus and then induce anterograde mechanisms to protect mitochondria and restore cellular homeostasis (Kasai *et al.*, 2019; Melber and Haynes, 2018). In mammals, several branches of the MRSP, including mitochondrial unfolded protein response (UPR^mt^), mitophagy, and the ISR pathway are known. However, many experimental paradigms indicate that the UPR^mt^ can be cross-regulated with ISR (Kasai *et al.*, 2019; Melber and Haynes, 2018). This review discusses the regulation and function of MRSP with a focus on the ISR pathway.

In the life cycle of a cell, transient inhibition of global protein synthesis prevents unwanted translation during mitosis (Sivan and Elroy-Stein, 2008). It is assumed that eIF2α phosphorylation can repress global translation during the G2/M phase (Kim *et al.*, 2018). However, which kinase is responsible for eIF2α phosphorylation and how an eIF2α kinase is activated during the mitotic phase are not fully understood. Kim *et al.* suggest that PKR is a responsible enzyme that can be activated by cellular double-stranded RNAs (dsRNAs) during mitosis (Kim *et al.*, 2018). PKR induces an immune response and phosphorylates eIF2α by sensing viral dsRNAs (Donnelly *et al.*, 2013; Wek *et al.*, 2006). However, PKR signaling is also assumed to be regulated by various cellular dsRNAs, such as mitochondrial RNAs (mtRNAs), inverted Alu repeats containing mRNAs (IRAlus mRNAs), and noncoding RNAs (ncRNAs) (Elbarbary *et al.*, 2013; Golec *et al.*, 2019; Kim *et al.*, 2018; Murad *et al.*, 2006). The ncRNAs and IRAlus mRNAs originate primarily in the nuclear genome but mtRNAs are found in the mitochondria genome. Kim *et al.* indicated that mtRNAs are a major class of PKR-interacting cellular dsRNAs and their increased expression may account for PKR phosphorylation during the mitotic phase (Kim *et al.*, 2018). They also suggested that PKR can localize in both cytosolic and mitochondrial regions and be activated by the mtRNAs in the mitochondria matrix under normal conditions, including the M phase (Kim *et al.*, 2018). However, Kim did not provide a mechanism to explain how PKR has mitochondria localization and PKR activated by mtRNAs phosphorylates cytosolic eIF2α. Besides PKR-eIF2α signaling activated by mtRNAs residing in mitochondria, regulation can be a result of mtRNAs released to the cytosol during stress conditions (Kim *et al.*, 2018). Treatment of two stress inducers, okadaic acid (OA, PKR phosphatase inhibitor) and staurosporine (STP, non-selective protein kinase inhibitor), leads to the cytosolic release of mtRNA by disruption of the mitochondrial membrane. Cytosolic interaction of PKR-mtRNA can then induce an immune response and eIF2α phosphorylation-mediated signaling, both of which can be used in beneficial ways. However, under stressful conditions during which PKR is overactivated by mitochondrial dysfunction, it is possible that mtRNA-mediated PKR activation can trigger inflammation and cell death (Rath *et al.*, 2012).

**Fig. 6:**
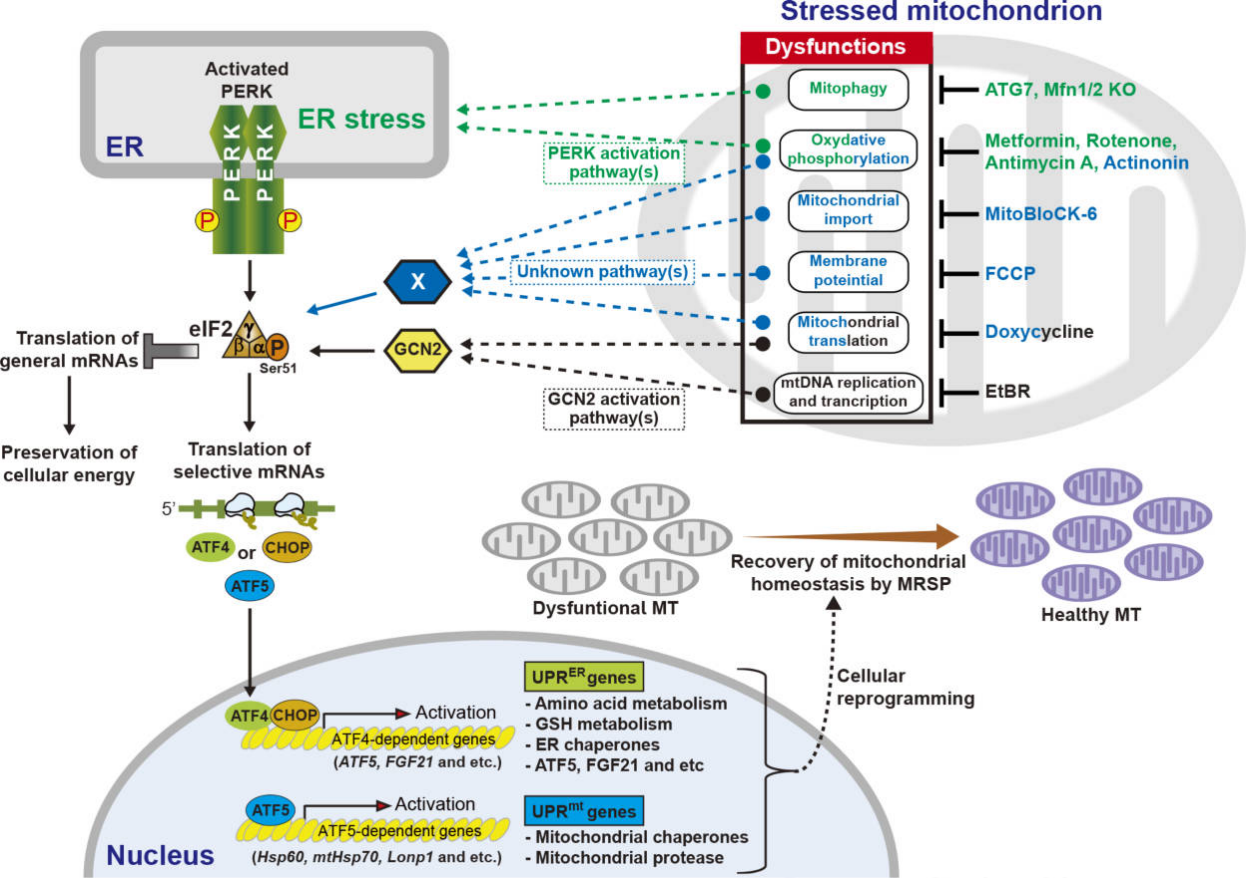
Mitochondrial retrograde signaling pathways (MRSP) induced by eIF2α phosphorylation in response to diverse mitochondrial stresses.

The MRSP is not limited to regulation of a cytosolic translation factor such as eIF2α phosphorylation but also extends to expression of several transcription factors to control nuclear gene expression (Kasai *et al.*, 2019; Melber and Haynes, 2018). In mammalian cells, CHOP in associated with C/EBPβ, regulates expression of UPR^mt^ genes (such as nuclear genes encoding Hsp60, Hsp10, mtDnaJ, and CIpP) in response to the accumulation of a deleted mutant form of the mitochondrial matrix protein, ornithine transcarbamylase (OTC) (Zhao *et al.*, 2002). ER unfolded protein stress response (UPR^ER^) is not involved in the CHOP-mediated gene expression of the UPR^mt^. Nevertheless, eIF2α-phosphorylation dependent ISR pathway reportedly can interact with multiple points of the UPR^mt^ (Fiorese *et al.*, 2016; Kasai *et al.*, 2019; Melber and Haynes, 2018; Quiros *et al.*, 2017). Other mitochondrial dysfunctions (mtDNA depletion by EtBr, ethidium bromide treatment, mitochondrial translation inhibition by doxycycline treatment) activate GCN2 and then the eIF2α phosphorylation-ATF4 pathway to induce UPR^ER^ including CHOP expression, but not UPR^mt^ (Michel *et al.*, 2015) (Fig. 6). Recently, the bZIP transcription factor ATF5 was proposed to regulate the mammalian UPR^mt^ pathway in a manner analogous to ATFS-1 in *Caenorhabditis elegans* (Fiorese *et al.*, 2016). The transcriptional expression of *ATF5* is induced by both ATF4 and CHOP and its translation is regulated by eIF2α phosphorylation (Teske *et al.*, 2013; Zhou *et al.*, 2008). The expression of ATF5 is responsible for upregulation of mitochondrial chaperone genes (Hsp60 and mtHsp70) and the protease gene (Lonp1) in responses to oxidative stress or oxidative phosphorylation inhibition (Fiorese *et al.*, 2016), which also induces an ER stress response (Wang *et al.*, 2018). These findings suggest that there are significant degrees of overlap in target gene regulation between the eIF2α-phosphorylation-dependent ISR pathway and the UPR^mt^. Furthermore, a multiomics analysis in HeLa cell mitochondrial stress models identified ATF4, which is a target translationally regulated by eIF2α phosphorylation, as a key regulator of stress response (Quiros *et al.*, 2017). All four different drugs that alter mitochondrial homeostasis in a different way (FCCP/proton ionophore, actinonin/mETC protein stability modifier, doxycycline/mitochondrial translation inhibitor, MitoBloCK-6/mitochondrial protein import inhibitor) activated the eIF2α phosphorylation–dependent ISR pathway. However, the ISR pathway activation was not dependent on the four known eIF2α kinases. Still, during mitochondrial stress, the ISR-ATF4 pathway activated the expression of many cytoprotective genes, including amino acid metabolism and GSH metabolism to maintain cell proliferation and provide protection against mitochondrial stress (Back and Kaufman, 2012; Quiros *et al.*, 2017) (Fig. 6). The IRS-ATF4 pathway is therefore an important player in MRSP, regulating both cytosolic translation and mitochondria homeostasis. However, upregulation of canonical UPR^mt^ genes mediated by ATF5 (Quiros *et al.*, 2017) has not been reported and it is possible that the ISR pathway in MRSP can be divided into ATF4-dependent and independent pathways, although further study is required.

Although the involvement of eIF2α kinases was not carefully studied in the conditions of the MRSP described above, data indicate that activated eIF2α kinases are responsible for eIF2α phosphorylation in mitochondrial stress conditions. Multiple studies have reported that an endocrine hormone, fibroblast growth factor 21 (FGF21), is expressed in an ATF4-dependent manner in response to mitochondria stresses induced by mETC inhibition, mitophagy defects, and defects in mitochondrial dynamics (Joe *et al.*, 2018; Kim *et al.*, 2013a; Kim *et al.*, 2013b). Metformin, which can inhibit mitochondrial complex 1, increased FGF21 expression via the ISR-ATF4 pathway activated by PERK (Kim *et al.*, 2013a). Metformin-induced ATF4 activation was inhibited by treatment with the mitochondrial antioxidant Mito-TEMPO, suggesting a role for the mitochondrial-derived ROS-PERK axis. In addition, rotenone (a mitochondrial complex I inhibitor) or antimycin A (a mitochondrial complex III inhibitor) also induced FGF21 gene expression via eIF2α phosphorylation–ATF4 activation in C2C12 myotubes (Kim *et al.*, 2013b). In muscle-specific KO mice of ATG7 or mitofusin 1 (Mfn1) and 2 (Mfn2), mitochondrial function was decreased by defects in mitophagy or mitochondrial dynamics, respectively (Kim *et al.*, 2013b). Mitochondrial dysfunctions were also responsible for FGF21 induction by eIF2α phosphorylation–ATF4 activation. These effects are most likely mediated by the activation of the PERK pathway because the knockout of ATG7 (Antonucci *et al.*, 2015; Yang *et al.*, 2010) or Mfn2 (Sebastian *et al.*, 2012) causes ER stress and activates PERK (Fig. 6). During mitochondrial stresses cells use eIF2α phosphorylation–mediated translation regulation and the ISR pathway to preserve cellular energy and restore mitochondrial homeostasis. However, an eIF2α phosphorylation–mediated response can produce unwanted and contradictory results. In *Drosophila melanogaster*, overexpression (O/E) of the mitochondrial mutant protein *prel/ups1p* induces dendritic regression of *Drosophila* peripheral sensory neurons by mitochondrial dysfunction (Tsuyama *et al.*, 2017). A mechanical study of cell regression revealed that *prel* O/E-mediated mitochondrial dysfunction induced prolonged translation suppression in neurons in a PERK-eIF2α phosphorylation–dependent manner, which led to dendrite loss, whereas it contributed to the maintenance of cellular ATP levels through translation inhibition (Tsuyama *et al.*, 2017). Collectively, these observations indicate that successful adaptation to cellular stress requires eIF2α phosphorylation to be in a balanced range (Wek and Anthony, 2009).

## Conclusions

Mammalian cells are equipped with four eIF2α kinases to sense and control diverse intracellular and extracellular stresses. The cells activate a common adaptive pathway, the ISR, through eIF2α phosphorylation, to restore cellular homeostasis, and eIF2α phosphorylation is involved in multiple biological processes. In protein synthesis, eIF2α phosphorylation suppresses translational initiation of general mRNAs and activates it from specific mRNAs in response to stress. However, regulation of protein synthesis by eIF2α phosphorylation does not always work as intended. In effect, too much is as bad as too little. Hyper and prolonged phosphorylation of eIF2α can increase expression of proapoptotic genes through downstream transcription factors, which can make matters worse, and sustained translational suppression can disrupt cellular homeostasis through deficiency of cell type–specific proteins or important cellular function–maintaining proteins. Beyond translation regulation, both hyper- and hypo-phosphorylation of eIF2α cause ROS damage because of both translational dysregulation of general mRNAs and the absence or overexpression of its downstream target genes. In addition, eIF2α phosphorylation can modulate mitochondrial structure and shape to maintain homeostasis and preserve its integrity through the PERK-eIF2α axis during ER stress. Mitochondria appear to use eIF2α phosphorylation to communicate with the cytosol and nucleus and protect and restore mitochondria homeostasis. Mitochondrial RNAs are recognized by PKR to regulate cytosolic translation through eIF2α phosphorylation during the mitotic phase and mitochondrial stress conditions. In mitochondria-nuclear communication, diverse mitochondrial dysfunctions, except for accumulation of a misfolded mitochondrial protein, activate eIF2α phosphorylation–dependent ISR pathways, which can be divided into ATF4-dependent and -independent pathways. However, only further study can shed light on what induces eIF2α phosphorylation during mitochondrial dysfunctions. MRSP induced by eIF2α phosphorylation does not always protect or ameliorate but can deteriorate mitochondria stress. The level of eIF2α phosphorylation should be in a balanced range to promote successful adaptation to cellular stress.

## References

[B1] Andreev, D.E., O’Connor, P.B., Fahey, C., Kenny, E.M., Terenin, I.M., Dmitriev, S.E., Cormican, P., Morris, D.W., Shatsky, I.N., and Baranov, P.V. 2015. Translation of 5' leaders is pervasive in genes resistant to eIF2 repression. Elife, 4: e03971. 25621764 10.7554/eLife.03971 PMC4383229

[B2] Antonucci, L., Fagman, J.B., Kim, J.Y., Todoric, J., Gukovsky, I., Mackey, M., Ellisman, M.H., and Karin, M. 2015. Basal autophagy maintains pancreatic acinar cell homeostasis and protein synthesis and prevents ER stress. Proc. Natl. Acad. Sci. USA, 112: E6166–6174. 26512112 10.1073/pnas.1519384112 PMC4653219

[B3] Back, S.H. and Kaufman, R.J. 2012. Endoplasmic reticulum stress and type 2 diabetes. Annu. Rev. Biochem., 81: 767–793. 22443930 10.1146/annurev-biochem-072909-095555 PMC3684428

[B4] Back, S.H., Scheuner, D., Han, J., Song, B., Ribick, M., Wang, J., Gildersleeve, R.D., Pennathur, S., and Kaufman, R.J. 2009. Translation attenuation through eIF2alpha phosphorylation prevents oxidative stress and maintains the differentiated state in beta cells. Cell Metab., 10: 13–26. 19583950 10.1016/j.cmet.2009.06.002 PMC2742645

[B5] Baird, T.D., Palam, L.R., Fusakio, M.E., Willy, J.A., Davis, C.M., McClintick, J.N., Anthony, T.G., and Wek, R.C. 2014. Selective mRNA translation during eIF2 phosphorylation induces expression of IBTKalpha. Mol. Biol. Cell, 25: 1686–1697. 24648495 10.1091/mbc.E14-02-0704 PMC4019499

[B6] Balsa, E., Soustek, M.S., Thomas, A., Cogliati, S., Garcia-Poyatos, C., Martin-Garcia, E., Jedrychowski, M., Gygi, S.P., Enriquez, J.A., and Puigserver, P. 2019. ER and Nutrient Stress Promote Assembly of Respiratory Chain Supercomplexes through the PERK-eIF2alpha Axis. Mol. Cell, 74: 877–890 e876. 31023583 10.1016/j.molcel.2019.03.031 PMC6555668

[B7] Brookes, P.S., Yoon, Y., Robotham, J.L., Anders, M.W., and Sheu, S.S. 2004. Calcium, ATP, and ROS: a mitochondrial love-hate triangle. Am. J. Physiol. Cell Physiol., 287: C817–833. 15355853 10.1152/ajpcell.00139.2004

[B8] Calkhoven, C.F., Muller, C., and Leutz, A. 2000. Translational control of C/EBPalpha and C/EBPbeta isoform expression. Genes. Dev., 14: 1920–1932. 10921906 PMC316813

[B9] Choi, W.G., Han, J., Kim, J.H., Kim, M.J., Park, J.W., Song, B., Cha, H.J., Choi, H.S., Chung, H.T., Lee, I.K., Park, T.S., Hatzoglou, M., Choi, H.S., Yoo, H.J., Kaufman, R.J., and Back, S.H. 2017. eIF2alpha phosphorylation is required to prevent hepatocyte death and liver fibrosis in mice challenged with a high fructose diet. Nutr. Metab. (Lond), 14: 48. 28781602 10.1186/s12986-017-0202-6 PMC5537942

[B10] Cogliati, S., Enriquez, J.A., and Scorrano, L. 2016. Mitochondrial Cristae: Where Beauty Meets Functionality. Trends. Biochem. Sci., 41: 261–273. 26857402 10.1016/j.tibs.2016.01.001

[B11] Donnelly, N., Gorman, A.M., Gupta, S., and Samali, A. 2013. The eIF2alpha kinases: their structures and functions. Cell. Mol. Life Sci., 70: 3493–3511. 23354059 10.1007/s00018-012-1252-6 PMC11113696

[B12] Elbarbary, R.A., Li, W., Tian, B., and Maquat, L.E. 2013. STAU1 binding 3' UTR IRAlus complements nuclear retention to protect cells from PKR-mediated translational shutdown. Genes. Dev., 27: 1495–1510. 23824540 10.1101/gad.220962.113 PMC3713430

[B13] Fernandez, J., Yaman, I., Sarnow, P., Snider, M.D., and Hatzoglou, M. 2002. Regulation of internal ribosomal entry site-mediated translation by phosphorylation of the translation initiation factor eIF2alpha. J. Biol. Chem., 277: 19198–19205. 11877448 10.1074/jbc.M201052200

[B14] Fiorese, C.J., Schulz, A.M., Lin, Y.F., Rosin, N., Pellegrino, M.W., and Haynes, C.M. 2016. The Transcription Factor ATF5 Mediates a Mammalian Mitochondrial UPR. Curr. Biol., 26: 2037–2043. 27426517 10.1016/j.cub.2016.06.002 PMC4980197

[B15] Fusakio, M.E., Willy, J.A., Wang, Y., Mirek, E.T., Al Baghdadi, R.J., Adams, C.M., Anthony, T.G., and Wek, R.C. 2016. Transcription factor ATF4 directs basal and stress-induced gene expression in the unfolded protein response and cholesterol metabolism in the liver. Mol. Biol. Cell, 27: 1536–1551. 26960794 10.1091/mbc.E16-01-0039 PMC4850040

[B16] Golec, E., Lind, L., Qayyum, M., Blom, A.M., and King, B.C. 2019. The Noncoding RNA nc886 Regulates PKR Signaling and Cytokine Production in Human Cells. J. Immunol., 202: 131–141. 30518569 10.4049/jimmunol.1701234

[B17] Gomes, L.C., Di Benedetto, G., and Scorrano, L. 2011. Essential amino acids and glutamine regulate induction of mitochondrial elongation during autophagy. Cell Cycle, 10: 2635–2639. 21811092 10.4161/cc.10.16.17002

[B18] Han, J., Back, S.H., Hur, J., Lin, Y.H., Gildersleeve, R., Shan, J., Yuan, C.L., Krokowski, D., Wang, S., Hatzoglou, M., Kilberg, M.S., Sartor, M.A., and Kaufman, R.J. 2013. ER-stress-induced transcriptional regulation increases protein synthesis leading to cell death. Nat. Cell Biol., 15: 481–490. 23624402 10.1038/ncb2738 PMC3692270

[B19] Harding, H.P., Zhang, Y., Zeng, H., Novoa, I., Lu, P.D., Calfon, M., Sadri, N., Yun, C., Popko, B., Paules, R., Stojdl, D.F., Bell, J.C., Hettmann, T., Leiden, J.M., and Ron, D. 2003. An integrated stress response regulates amino acid metabolism and resistance to oxidative stress. Mol. Cell, 11: 619–633. 12667446 10.1016/s1097-2765(03)00105-9

[B20] Harding, H.P., Zhang, Y., Scheuner, D., Chen, J.J., Kaufman, R.J., and Ron, D. 2009. Ppp1r15 gene knockout reveals an essential role for translation initiation factor 2 alpha (eIF2alpha) dephosphorylation in mammalian development. Proc. Natl. Acad. Sci. USA, 106: 1832–1837. 19181853 10.1073/pnas.0809632106 PMC2644123

[B21] Hinnebusch, A.G., Ivanov, I.P., and Sonenberg, N. 2016. Translational control by 5'-untranslated regions of eukaryotic mRNAs. Science, 352: 1413–1416. 27313038 10.1126/science.aad9868 PMC7422601

[B22] Hussain, S.G. and Ramaiah, K.V. 2007. Reduced eIF2alpha phosphorylation and increased proapoptotic proteins in aging. Biochem. Biophys. Res. Commun., 355: 365–370. 17300747 10.1016/j.bbrc.2007.01.156

[B23] Iurlaro, R. and Munoz-Pinedo, C. 2016. Cell death induced by endoplasmic reticulum stress. FEBS J., 283: 2640–2652. 26587781 10.1111/febs.13598

[B24] Joe, Y., Kim, S., Kim, H.J., Park, J., Chen, Y., Park, H.J., Jekal, S.J., Ryter, S.W., Kim, U.H., and Chung, H.T. 2018. FGF21 induced by carbon monoxide mediates metabolic homeostasis via the PERK/ATF4 pathway. FASEB J., 32: 2630–2643. 29295856 10.1096/fj.201700709RR PMC5901375

[B25] Kasai, S., Yamazaki, H., Tanji, K., Engler, M.J., Matsumiya, T., and Itoh, K. 2019. Role of the ISR-ATF4 pathway and its cross talk with Nrf2 in mitochondrial quality control. J. Clin. Biochem. Nutr., 64: 1–12. 30705506 10.3164/jcbn.18-37 PMC6348405

[B26] Kilberg, M.S., Shan, J., and Su, N. 2009. ATF4-dependent transcription mediates signaling of amino acid limitation. Trends Endocrinol. Metab., 20: 436–443. 19800252 10.1016/j.tem.2009.05.008 PMC3587693

[B27] Kim, K.H., Jeong, Y.T., Kim, S.H., Jung, H.S., Park, K.S., Lee, H.Y., and Lee, M.S. 2013a. Metformin-induced inhibition of the mitochondrial respiratory chain increases FGF21 expression via ATF4 activation. Biochem. Biophys. Res. Commun., 440: 76–81. 24041694 10.1016/j.bbrc.2013.09.026

[B28] Kim, K.H., Jeong, Y.T., Oh, H., Kim, S.H., Cho, J.M., Kim, Y.N., Kim, S.S., Kim, D.H., Hur, K.Y., Kim, H.K., Ko, T., Han, J., Kim, H.L., Kim, J., Back, S.H., Komatsu, M., Chen, H., Chan, D.C., Konishi, M., Itoh, N., Choi, C.S., and Lee, M.S. 2013b. Autophagy deficiency leads to protection from obesity and insulin resistance by inducing Fgf21 as a mitokine. Nat. Med., 19: 83–92. 23202295 10.1038/nm.3014

[B29] Kim, Y., Park, J., Kim, S., Kim, M., Kang, M.G., Kwak, C., Kang, M., Kim, B., Rhee, H.W., and Kim, V.N. 2018. PKR Senses Nuclear and Mitochondrial Signals by Interacting with Endogenous Double-Stranded RNAs. Mol. Cell, 71: 1051–1063 e1056. 30174290 10.1016/j.molcel.2018.07.029

[B30] Lapuente-Brun, E., Moreno-Loshuertos, R., Acin-Perez, R., Latorre-Pellicer, A., Colas, C., Balsa, E., Perales-Clemente, E., Quiros, P.M., Calvo, E., Rodriguez-Hernandez, M.A., Navas, P., Cruz, R., Carracedo, A., Lopez-Otin, C., Perez-Martos, A., Fernandez-Silva, P., Fernandez-Vizarra, E., and Enriquez, J.A. 2013. Supercomplex assembly determines electron flux in the mitochondrial electron transport chain. Science, 340: 1567–1570. 23812712 10.1126/science.1230381

[B31] Lebeau, J., Saunders, J.M., Moraes, V.W.R., Madhavan, A., Madrazo, N., Anthony, M.C., and Wiseman, R.L. 2018. The PERK Arm of the Unfolded Protein Response Regulates Mitochondrial Morphology during Acute Endoplasmic Reticulum Stress. Cell Rep., 22: 2827–2836. 29539413 10.1016/j.celrep.2018.02.055 PMC5870888

[B32] Lee, Y.Y., Cevallos, R.C., and Jan, E. 2009. An upstream open reading frame regulates translation of GADD34 during cellular stresses that induce eIF2alpha phosphorylation. J. Biol. Chem., 284: 6661–6673. 19131336 10.1074/jbc.M806735200 PMC2652341

[B33] Lewerenz, J. and Maher, P. 2009. Basal levels of eIF2alpha phosphorylation determine cellular antioxidant status by regulating ATF4 and xCT expression. J. Biol. Chem., 284: 1106–1115. 19017641 10.1074/jbc.M807325200 PMC2613630

[B34] Li, G., Mongillo, M., Chin, K.T., Harding, H., Ron, D., Marks, A.R., and Tabas, I. 2009. Role of ERO1-alpha-mediated stimulation of inositol 1,4,5-triphosphate receptor activity in endoplasmic reticulum stress-induced apoptosis. J. Cell Biol., 186: 783–792. 19752026 10.1083/jcb.200904060 PMC2753154

[B35] Li, Y., Bevilacqua, E., Chiribau, C.B., Majumder, M., Wang, C., Croniger, C.M., Snider, M.D., Johnson, P.F., and Hatzoglou, M. 2008. Differential control of the CCAAT/enhancer-binding protein beta (C/EBPbeta) products liver-enriched transcriptional activating protein (LAP) and liver-enriched transcriptional inhibitory protein (LIP) and the regulation of gene expression during the response to endoplasmic reticulum stress. J. Biol. Chem., 283: 22443–22456. 18550528 10.1074/jbc.M801046200 PMC2504880

[B36] Maillo, C., Martin, J., Sebastian, D., Hernandez-Alvarez, M., Garcia-Rocha, M., Reina, O., Zorzano, A., Fernandez, M., and Mendez, R. 2017. Circadian- and UPR-dependent control of CPEB4 mediates a translational response to counteract hepatic steatosis under ER stress. Nat. Cell Biol., 19: 94–105. 28092655 10.1038/ncb3461

[B37] Melber, A. and Haynes, C.M. 2018. UPR(mt) regulation and output: a stress response mediated by mitochondrial-nuclear communication. Cell Res., 28: 281–295. 29424373 10.1038/cr.2018.16 PMC5835775

[B38] Michel, S., Canonne, M., Arnould, T., and Renard, P. 2015. Inhibition of mitochondrial genome expression triggers the activation of CHOP-10 by a cell signaling dependent on the integrated stress response but not the mitochondrial unfolded protein response. Mitochondrion, 21: 58–68. 25643991 10.1016/j.mito.2015.01.005

[B39] Moreno, J.A., Radford, H., Peretti, D., Steinert, J.R., Verity, N., Martin, M.G., Halliday, M., Morgan, J., Dinsdale, D., Ortori, C.A., Barrett, D.A., Tsaytler, P., Bertolotti, A., Willis, A.E., Bushell, M., and Mallucci, G.R. 2012. Sustained translational repression by eIF2alpha-P mediates prion neurodegeneration. Nature, 485: 507–511. 22622579 10.1038/nature11058 PMC3378208

[B40] Munoz, J.P., Ivanova, S., Sanchez-Wandelmer, J., Martinez-Cristobal, P., Noguera, E., Sancho, A., Diaz-Ramos, A., Hernandez-Alvarez, M.I., Sebastian, D., Mauvezin, C., Palacin, M., and Zorzano, A. 2013. Mfn2 modulates the UPR and mitochondrial function via repression of PERK. EMBO J., 32: 2348–2361. 23921556 10.1038/emboj.2013.168 PMC3770335

[B41] Murad, J.M., de Souza, L.R., and De Lucca, F.L. 2006. PKR activation by a non-coding RNA expressed in lymphocytes of mice bearing B16 melanoma. Blood Cells Mol. Dis., 37: 128–133. 16857398 10.1016/j.bcmd.2006.05.004

[B42] Pakos-Zebrucka, K., Koryga, I., Mnich, K., Ljujic, M., Samali, A., and Gorman, A.M. 2016. The integrated stress response. EMBO Rep., 17: 1374–1395. 27629041 10.15252/embr.201642195 PMC5048378

[B43] Palam, L.R., Baird, T.D., and Wek, R.C. 2011. Phosphorylation of eIF2 facilitates ribosomal bypass of an inhibitory upstream ORF to enhance CHOP translation. J. Biol. Chem., 286: 10939–10949. 21285359 10.1074/jbc.M110.216093 PMC3064149

[B44] Proud, C.G. 2005. eIF2 and the control of cell physiology. Semin. Cell Dev. Biol., 16: 3–12. 15659334 10.1016/j.semcdb.2004.11.004

[B45] Quiros, P.M., Prado, M.A., Zamboni, N., D’Amico, D., Williams, R.W., Finley, D., Gygi, S.P., and Auwerx, J. 2017. Multi-omics analysis identifies ATF4 as a key regulator of the mitochondrial stress response in mammals. J. Cell Biol., 216: 2027–2045. 28566324 10.1083/jcb.201702058 PMC5496626

[B46] Rajesh, K., Papadakis, A.I., Kazimierczak, U., Peidis, P., Wang, S., Ferbeyre, G., Kaufman, R.J., and Koromilas, A.E. 2013. eIF2alpha phosphorylation bypasses premature senescence caused by oxidative stress and pro-oxidant antitumor therapies. Aging (Albany NY), 5: 884–901. 24334569 10.18632/aging.100620 PMC3883705

[B47] Rambold, A.S., Kostelecky, B., Elia, N., and Lippincott-Schwartz, J. 2011. Tubular network formation protects mitochondria from autophagosomal degradation during nutrient starvation. Proc. Natl. Acad. Sci. USA, 108: 10190–10195. 21646527 10.1073/pnas.1107402108 PMC3121813

[B48] Rath, E., Berger, E., Messlik, A., Nunes, T., Liu, B., Kim, S.C., Hoogenraad, N., Sans, M., Sartor, R.B., and Haller, D. 2012. Induction of dsRNA-activated protein kinase links mitochondrial unfolded protein response to the pathogenesis of intestinal inflammation. Gut, 61: 1269–1278. 21997551 10.1136/gutjnl-2011-300767 PMC4514769

[B49] Ron, D. 2002. Translational control in the endoplasmic reticulum stress response. J. Clin. Invest., 110: 1383–1388. 12438433 10.1172/JCI16784 PMC151821

[B50] Ron, D. and Walter, P. 2007. Signal integration in the endoplasmic reticulum unfolded protein response. Nat. Rev. Mol. Cell Biol., 8: 519–529. 17565364 10.1038/nrm2199

[B51] Sato, H., Nomura, S., Maebara, K., Sato, K., Tamba, M., and Bannai, S. 2004. Transcriptional control of cystine/glutamate transporter gene by amino acid deprivation. Biochem. Biophys. Res. Commun., 325: 109–116. 15522208 10.1016/j.bbrc.2004.10.009

[B52] Scheuner, D., Song, B., McEwen, E., Liu, C., Laybutt, R., Gillespie, P., Saunders, T., Bonner-Weir, S., and Kaufman, R.J. 2001. Translational control is required for the unfolded protein response and in vivo glucose homeostasis. Mol. Cell, 7: 1165–1176. 11430820 10.1016/s1097-2765(01)00265-9

[B53] Sebastian, D., Hernandez-Alvarez, M.I., Segales, J., Sorianello, E., Munoz, J.P., Sala, D., Waget, A., Liesa, M., Paz, J.C., Gopalacharyulu, P., Oresic, M., Pich, S., Burcelin, R., Palacin, M., and Zorzano, A. 2012. Mitofusin 2 (Mfn2) links mitochondrial and endoplasmic reticulum function with insulin signaling and is essential for normal glucose homeostasis. Proc. Natl. Acad. Sci. USA, 109: 5523–5528. 22427360 10.1073/pnas.1108220109 PMC3325712

[B54] Sivan, G. and Elroy-Stein, O. 2008. Regulation of mRNA Translation during cellular division. Cell Cycle, 7: 741–744. 18239464 10.4161/cc.7.6.5596

[B55] Sonenberg, N. and Hinnebusch, A.G. 2009. Regulation of translation initiation in eukaryotes: mechanisms and biological targets. Cell, 136: 731–745. 19239892 10.1016/j.cell.2009.01.042 PMC3610329

[B56] Tabas, I. and Ron, D. 2011. Integrating the mechanisms of apoptosis induced by endoplasmic reticulum stress. Nat. Cell Biol., 13: 184–190. 21364565 10.1038/ncb0311-184 PMC3107571

[B57] Taniuchi, S., Miyake, M., Tsugawa, K., Oyadomari, M., and Oyadomari, S. 2016. Integrated stress response of vertebrates is regulated by four eIF2alpha kinases. Sci. Rep., 6: 32886. 27633668 10.1038/srep32886 PMC5025754

[B58] Teske, B.F., Fusakio, M.E., Zhou, D., Shan, J., McClintick, J.N., Kilberg, M.S., and Wek, R.C. 2013. CHOP induces activating transcription factor 5 (ATF5) to trigger apoptosis in response to perturbations in protein homeostasis. Mol. Biol. Cell, 24: 2477–2490. 23761072 10.1091/mbc.E13-01-0067 PMC3727939

[B59] Tsuyama, T., Tsubouchi, A., Usui, T., Imamura, H., and Uemura, T. 2017. Mitochondrial dysfunction induces dendritic loss via eIF2alpha phosphorylation. J. Cell. Biol., 216: 815–834. 28209644 10.1083/jcb.201604065 PMC5346966

[B60] Wang, M. and Kaufman, R.J. 2014. The impact of the endoplasmic reticulum protein-folding environment on cancer development. Nat. Rev. Cancer, 14: 581–597. 25145482 10.1038/nrc3800

[B61] Wang, R., Sun, D.Z., Song, C.Q., Xu, Y.M., Liu, W., Liu, Z., and Dong, X.S. 2018. Eukaryotic translation initiation factor 2 subunit alpha (eIF2alpha) inhibitor salubrinal attenuates paraquat-induced human lung epithelial-like A549 cell apoptosis by regulating the PERK-eIF2alpha signaling pathway. Toxicol. In Vitro, 46: 58–65. 28986289 10.1016/j.tiv.2017.10.006

[B62] Wek, R.C., Jiang, H.Y., and Anthony, T.G. 2006. Coping with stress: eIF2 kinases and translational control. Biochem. Soc. Trans., 34: 7–11. 16246168 10.1042/BST20060007

[B63] Wek, R.C. and Anthony, T.G. 2009. Beta testing the antioxidant function of eIF2alpha phosphorylation in diabetes prevention. Cell Metab., 10: 1–2. 19583945 10.1016/j.cmet.2009.06.005

[B64] Wortham, N.C. and Proud, C.G. 2015. eIF2B: recent structural and functional insights into a key regulator of translation. Biochem. Soc. Trans., 43: 1234–1240. 26614666 10.1042/BST20150164

[B65] Yang, L., Li, P., Fu, S., Calay, E.S., and Hotamisligil, G.S. 2010. Defective hepatic autophagy in obesity promotes ER stress and causes insulin resistance. Cell Metab., 11: 467–478. 20519119 10.1016/j.cmet.2010.04.005 PMC2881480

[B66] Young, S.K., Baird, T.D., and Wek, R.C. 2016. Translation Regulation of the Glutamyl-prolyl-tRNA Synthetase Gene EPRS through Bypass of Upstream Open Reading Frames with Noncanonical Initiation Codons. J. Biol. Chem., 291: 10824–10835. 27002157 10.1074/jbc.M116.722256 PMC4865927

[B67] Young, S.K. and Wek, R.C. 2016. Upstream Open Reading Frames Differentially Regulate Gene-specific Translation in the Integrated Stress Response. J. Biol. Chem., 291: 16927–16935. 27358398 10.1074/jbc.R116.733899 PMC5016099

[B68] Zhao, Q., Wang, J., Levichkin, I.V., Stasinopoulos, S., Ryan, M.T., and Hoogenraad, N.J. 2002. A mitochondrial specific stress response in mammalian cells. EMBO J., 21: 4411–4419. 12198143 10.1093/emboj/cdf445 PMC126185

[B69] Zheng, M., Kim, S.K., Joe, Y., Back, S.H., Cho, H.R., Kim, H.P., Ignarro, L.J., and Chung, H.T. 2012. Sensing endoplasmic reticulum stress by protein kinase RNA-like endoplasmic reticulum kinase promotes adaptive mitochondrial DNA biogenesis and cell survival via heme oxygenase-1/carbon monoxide activity. FASEB J., 26: 2558–2568. 22391129 10.1096/fj.11-199604

[B70] Zhou, D., Palam, L.R., Jiang, L., Narasimhan, J., Staschke, K.A., and Wek, R.C. 2008. Phosphorylation of eIF2 directs ATF5 translational control in response to diverse stress conditions. J. Biol. Chem., 283: 7064–7073. 18195013 10.1074/jbc.M708530200

[B71] Zhu, P.J., Khatiwada, S., Cui, Y., Reineke, L.C., Dooling, S.W., Kim, J.J., Li, W., Walter, P., and Costa-Mattioli, M. 2019. Activation of the ISR mediates the behavioral and neurophysiological abnormalities in Down syndrome. Science, 366: 843–849. 31727829 10.1126/science.aaw5185 PMC7299149

